# Botany in Its Relations with Medicine—No. 5

**Published:** 1878-04

**Authors:** W. T. Grant

**Affiliations:** Atlanta, Ga.


Medical and Surgical Journal.

VOL. XVI.] APRIL—1878. [No. 1

### Original Communications.

### BOTANY IN ITS RELATIONS WITH MEDICINE.—5.

By W. T. GRANT, M.D., Atlanta, Ga.

Every one in eating an orange, or lemon, or pomegranate, or a slice of watermelon, must have observed in the pulp of these fruits the small, egg-like bodies containing the juice peculiar to each, and constituting the bulk of the fruit. These small bodies are specimens of the vegetable cell. All vegetable cells are essentially the same, whatever their position in the plant; that is, they all have two coats, one just within the other, and containing some one or other material, according to the purpose of the cell.

I will illustrate the cell with the common hen's egg. The egg-shell is the common outer coat of the cell; the thin membrane just inside the shell (which every one has seen on the hard-boiled egg) corresponds to the inner membrane; while the white of the egg and the yolk are simply the analogues of the contents of the cell, whatever these contents may be.

The vegetable cell is nature's great laboratory. Within the cell take place all those wonderful chemical operations of decomposition and recomposition, by which are produced all the various vegetable products. The vegetable cell, therefore, is the factory into which is carried the raw material, and wherein is generated from this material all the food of all animated nature, as well as the poisons and medicines and various other products obtained from or found in plants. The various elements of the mineral kingdom, both simple and compound, enter the vegetable cell as such; and by some inscrutable power, not now understood, they are variously combined and recombined, so that in a short time they have wholly lost their mineral character, and have become organized proximate compounds, having organization, and often endowed with vitality. All these wonderful changes take place in the vegetable cell. Hence, we are authorized to say that the opium of the poppy, the turpentine of the pine, the sugar of the beet and the cane, the quinine of the cinchonas, the starch of the potato, the gluten of wheat, and the thousands of other products, are generated or created in the cells of these various plants. And these cells, after a time, become filled with such products, each with its own particular product; and thus these products are retained and held in the cells where they are generated.

I will now call attention to two very important points of differences between vegetable and animal physiology, in this connection. In plants there is no such thing known as the "waste or effete material," which constantly enters the blood of animals as the result of various changes taking place in the tissues. Nor is there anything in the plant to correspond with it. The second point of difference is as follows: As the opium of the poppy, for instance, is produced in the cells, and as this act in the plant is the exact analogue of secretion in the animal body, we see that, while the secretory organs or glands in the animal are conglomerate, in the plant they are just the reverse—that is, diffuse; and thus we understand how it is that the peculiar product of the plant is scattered through the entire plant, because its secretory gland, consisting of simple cells, each performing its functions, is diffused as cells through all parts of the plant, and are not gathered together in one place as in the animal.

My purpose in what I have said above is now attained, which was to show how our quinine, opium, and other vegetable medicines, originate. They are as essentially secretions as are bile, or saliva, or urine. Another important point in this connection is this: That while all the animal secretions are produced from the "waste or effete material" of the body, and consequently participate in the degraded character of this material, which is in one stage of progressive degradation from a higher to a lower condition, the secretions of the vegetable is in the opposite condition—that is, they occupy a place in a progressive or advancing series of changes from a lower to a higher condition.

I cannot say that the thoughts now suggested are at present of much value to the physician or therapeutist, but I believe it is well to note them, as they will very certainly, at some future time, form a very important item in the progress of therapeutics, and consequently in the practice of medicine.

For some years past it has been a favorite opinion with me, that every medicinal plant that grows *has some physical and visible character, or combination of characters, which will indicate that it does possess such medical virtues; and that the same physical characters, or certain others, will indicate what* SPECIAL KIND *of virtue it possesses.* If this proposition could be established, then our great object as medical botanists would be to determine what characters would serve this purpose as indices. I have no hesitation in saying that our present botanical classifications would not afford the least aid in such a work. And it will be necessary for botanists, who shall at the same time be physlcians, to go over again the entire field of practical and descriptive botany. As evidence of the truth of what I say, I state that in present classifications the deadly nightshade (solanum nigrum) and the common sweet potatoe (solanum tuberosum) stand to each other in about the same relation as brother and sister; and other similar examples can be given, as in the genus rhus. Here we have the rhus glabra, or old-field sumach, an innocent plant, in immediate relationship with the rhus toxicodendron, or as it is commonly called, the poison-oak vine, a very dangerous plant.

It may be thought that this would be almost endless work to thus go over the entire field of descriptive botany, and take a new and exhaustive description of all plants. But that is not so. And if it were far more laborious than it will prove when tested, that very fact furnishes another strong argument in favor of the proposition which I have been urging for some years, that our medical colleges should each establish a chair of botany, so that their graduates may aid in this good work. And, as evidence that I am not exaggerating the facilities which I speak about, I have no hesitation in saying that I can take any man with ordinary abilities, and a fair education and in one year's time I will make him a good, fair practical botanist, fully competent to pursue the study without further aid; and this can be done without losing much time from any of the ordinary pursuits of life. How much easier then could it be done with students who are giving their whole time to the acquisition of the profession of medicine?

I will quote the following paragraphs from an article which I published some years since on this subject, as I do not think I can express the ideas in better form:

"If we were in the woods, and find a strange plant which has attracted our attention, how shall we know whether or not it is poisonous? Or whether it is a valuable or a worthless plant? I have not the least doubt that there is some arrangement of some of its parts which would tell us, if we could only interpret them correctly. The particular plan or color of the flower; the arrangement of the leaves; the number or form of the stamens, or petals, or pistils, or indeed any one of the infinite variations which any plant may exhibit, would be to us the desired index, if we could but read it aright.

"The variations among the physical characters of plants are nature's landmarks. And the very *least* variations, even of form or color, has a purpose, and *may* possess a value not now dreamed of in our philosophy. There is a beautiful net-work of laws interwoven through all plant life, and comes far more closely in contact with the rough exterior physical characters of plants than we imagine. Indeed, those physical characters are but the outgrowth, or outward manifestation of interior laws. These physical characters thus become the alphabet of plant life, or rather of vegetable therapeutics; and if combined into words and sentences, will constitute the language in which nature has written these laws. And if we can but learn to read it, we have before us in every plant we find, a manuscript wherein we may read clearly and distinctly, some of nature's profoundest secrets. And in these indices we may become possessed of the key which will open to our view the entire field of vegetable therapeutics, and the practice of medicine would become simply the application of a few plain rules, and we could foresee the course to pursue in any given case, as surely as we now do in working out a sum under the rule of three.

"To determine the different indices and their value, will require in an investigator an accurate knowledge of practical botany, together with a thorough knowledge of physiology and therapeutics. A botanist, however well informed he may be in botany, is not properly prepared to study this subject. It thus appears that the study of botany should be combined with that of medicine before we can have properly prepared investigators in the field. Consequently there is an absolute necessity that there should be a chair of botany in every medical college."

In conclusion, I will offer some suggestions to those who may take an interest in these subjects, which I think I have before published, but am not certain. These suggestions must be taken as true in a very general sense, and necessarily have more or less exceptions. I offer them as problems requiring solution, rather than definite truths.

Any plant having an umbeliflorate inflorescence is dangerous, and generally drastic in its effects.

The powder in the common mushroom, known as the puff-ball, is said to be a powerful and instantaneous styptic.

The excessive use of starchy food is said to injure the eye-sight.

Is it not true that the food produced in any country is better suited than imported food for preserving the health and vigor of the inhabitants of that country?

Is it not also reasonable to believe that every country produces an antidote for every disease which is native to the country?

An indefinite increase or multiplication of any part of a plant is an evidence of safety; *i. e.*, the rose, which has an indefinite number of petals and stamens.

Coffee is said to be a very reliable antidote for strychnine, and has cured dogs poisoned by it.

Plants in which the color of the flowers is not single and uniform, and which therefore varies, are innocent.

Plants having bulbous, cormose or rhizomatose roots are generally violent in their effects. Fibrous rooted plants are not generally very active, unless they have some irregularity in the flower. Coraline, sub-ærial, tuberous and tap-rooted plants are usually innocent or mild in their effects.

